# Impact of Ambient Temperature Sample Storage on the Equine Fecal Microbiota

**DOI:** 10.3390/ani11030819

**Published:** 2021-03-15

**Authors:** Michelle Martin de Bustamante, Caryn Plummer, Jennifer MacNicol, Diego Gomez

**Affiliations:** 1 Department of Small Animal Clinical Sciences, College of Veterinary Medicine, University of Florida, Gainesville, FL 32610, USA; michellemartinde@ufl.edu (M.M.d.B.); plummerc@ufl.edu (C.P.); 2 Department of Large Animal Clinical Sciences, College of Veterinary Medicine, University of Florida, Gainesville, FL 32610, USA; 3 Department of Animal Biosciences, Ontario Agriculture College, University of Guelph, Guelph, ON N1G 2W1, Canada; jmacnico@uoguelph.ca; 4 Department of Clinical Studies, Ontario Veterinary College, University of Guelph, Guelph, ON N1G 2W1, Canada

**Keywords:** fecal microbiota, high-throughput sequencing, horse, microbiome, storage, temperature

## Abstract

**Simple Summary:**

Sample storage technique may impact fecal bacterial microbiota composition (the collective community of bacteria present in feces). This is an especially important factor in field studies, where access to freezing or refrigeration may be limited or non-existent, resulting in samples remaining at room temperature until transport to the laboratory. The objective of this study was to investigate the effect of sample storage at room temperature for up to 96 h on the fecal microbiota of healthy horses. Results revealed that storage of equine fecal samples at room temperature for up to 6 h before freezing had minimal effect on the fecal microbiota, while longer term storage at room temperature led to alterations in the resident bacterial population. When ultra-low temperature storage conditions are unavailable for immediate freezing, equine fecal samples should be frozen within 6 h after collection to minimize storage induced alterations in bacterial composition.

**Abstract:**

Sample storage conditions are an important factor in fecal microbiota analyses in general. The objective of this study was to investigate the effect of sample storage at room temperature on the equine fecal microbiota composition. Fecal samples were collected from 11 healthy horses. Each sample was divided into 7 sealed aliquots. One aliquot was immediately frozen at −80 °C; the remaining aliquots were stored at room temperature (21 to 22 °C) with one transferred to the freezer at each of the following time points: 6, 12, 24, 48, 72 and 96 h. The Illumina MiSeq sequencer was used for high-throughput sequencing of the V4 region of the 16S rRNA gene. Fibrobacteraceae (*Fibrobacter*) and Ruminococcaceae (*Ruminococcus*) were enriched in samples from 0 h and 6 h, whereas taxa from the families Bacillaceae, Planococcaceae, Enterobacteriaceae and Moraxellaceae were enriched in samples stored at room temperature for 24 h or greater. Samples frozen within the first 12 h after collection shared similar community membership. The community structure was similar for samples collected at 0 h and 6 h, but it was significantly different between samples frozen at 0 h and 12 h or greater. In conclusion, storage of equine fecal samples at ambient temperature for up to 6 h before freezing following sample collection had minimal effect on the microbial composition. Longer-term storage at ambient temperature resulted in alterations in alpha-diversity, community membership and structure and the enrichment of different taxa when compared to fecal samples immediately frozen at −80 °C.

## 1. Introduction

The gastrointestinal (GI) microbiota consists of a community of microbial organisms, including bacteria, archaea, fungi and viruses, colonizing the GI tract. The GI microbiota is an increasingly important area of study in both human and veterinary medicine, as it has been demonstrated to play an important role in digestion, nutrient absorption, short chain fatty acid production, immune function, health and homeostasis [1,2]. Assessment of the fecal microbiota may be impacted by several exogenous factors, including fecal sample collection method, sample storage conditions and DNA extraction technique [3,4,5]. Sample storage conditions are an especially important consideration since horses may be located far from the laboratory setting making, resulting in a prolonged delay between sample collection and submission for analysis. Fecal samples used in next-generation sequencing techniques are traditionally frozen at −80 °C, as this is the gold standard for preservation of microbiota composition and samples may be stored for months before processing [5,6]. In a field setting, there may be limited access to a freezer or alternative cooling methods and samples may be left at room temperature (20 to 22 °C) [7] for a period of time before reaching the laboratory [8,9]. At ambient temperature following defecation, the microbiota present in a fecal sample is subject to oxygen and other environmental conditions, which may alter the relative abundance of different bacterial phyla [3,10,11].

The effect of ambient temperature on the human fecal microbiota has been assessed in several studies with conflicting results; some investigations found that sample storage in ambient temperature had no significant effect on microbiota diversity or composition [12,13], while others found that fecal microbiota composition changed significantly when stored short-term at ambient temperature [3]. Relevant studies in veterinary species are limited [14,15,16]; storage of porcine fecal samples at ambient temperature for 3 h resulted in no significant differences in DNA quality, yield or microbiota richness when compared to samples snap frozen in liquid nitrogen [16]. Storage of feline fecal samples at ambient temperature for up to four days had no significant effect on the relative bacterial abundance of the fecal microbiota, neither on the alpha or beta-diversity measurements when compared to control samples frozen within 1 h after defecation [8]. In horses, the relative abundance and bacterial composition of the main taxa remained stable for up to 6 h and 12 h of exposure to an ambient temperature between 10 to 20 °C or an average of 32 °C (SD ± 3.6 °C), respectively [14,15]. To the best of the authors’ knowledge, the effect of storage of fecal samples at room temperature beyond 24 h on the equine fecal microbiota has not been previously described in the literature. Given the conflicting results found in studies from different species, further investigation into the impact of ambient temperature on the fecal microbiota of horses is warranted in order to establish appropriate short-term storage protocols for field studies. The objective of this study was to investigate the effect of sample storage at room temperature for up to 96 h on the equine fecal microbiota composition. We hypothesized that the equine feal bacterial microbiota would not be significantly affected by sample storage at ambient temperature for up to 96 h prior to sample processing.

## 2. Materials and Methods

### 2.1. Ethical Considerations

This study was performed in accordance with the guidelines of the Institutional Animal Care and Use Committee of the University of Florida (IACUC #201910672).

### 2.2. Animals

Eleven healthy, adult horses from the University of Florida, College of Veterinary Medicine Equine Research Program (ERP) Shared Herd were enrolled in the study. At the time of enrollment, all horses received complete ophthalmic examinations under standing sedation by a board-certified veterinary ophthalmologist and ophthalmology resident to ensure they were free of ophthalmic disease. Additionally, each horse had a general physical examination performed by a board-certified veterinary internal medicine specialist to ensure they were systemically healthy. Fecal samples were obtained per rectum once from each enrolled horse using lubrication and disposable rectal sleeves. The study population consisted of 6 Thoroughbred mares and 5 Thoroughbred geldings between the ages of 4 and 12 years old (median 6.5 year). Patient breed, age, sex and body weight are detailed in Table 1. The horses were not in training. Standardized feeding practices were used for enrolled horses and all study participants were in pasture, supplemented with hay and grain. At the time of sample collection, the horses were kept in paddocks at the same facility in small groups of between 2 and 4 horses each for an unrelated study.

### 2.3. Sample Size Calculation

The sample size of 11 horses was based upon a previously published equine fecal microbiota study in which an a priori power calculation determined that a minimum sample size of 6 was necessary for a power of 0.80 with an alpha of 0.05 to detect a 25% change in operational taxonomic unit (OTU) counts, assuming a normal distribution with a mean ± SD OTU count of 2886 ± 391 per sample [17]. This sample size is also supported by the results of additional equine studies in which samples sizes of 6 to 7 animals per group (with paired controls) were sufficient to yield significant differences in the relative abundance of the bacterial microbiota at all taxonomic levels (from phyla to genus level) and diversity indices [18,19].

### 2.4. Sample Storage and Processing

Individual fecal samples were placed in sterile plastic 4 oz (118 mL) fecal containers and stored at ambient temperature with the lids secured until arrival at the laboratory (within 1 h of sample collection). Each fecal sample was then aseptically divided into 7 sealed aliquots of 2–3 g feces, each stored in individual 4 oz (118 mL) plastic screw top cap sterile specimen containers. One aliquot was immediately frozen at −80 °C; the remaining 6 aliquots were stored at room temperature (range 21 to 22 °C), with one aliquot transferred to the −80 °C freezer at each of the following time points: 6, 12, 24, 48, 72 and 96 h following arrival at the laboratory. Thereafter, all samples remained in the −80 °C freezer until processing. At the time of processing, all samples were thawed and immediately thereafter bacterial DNA was extracted using a commercially available kit (EZNA Stool DNA Kit) following manufacturer recommendations. Following extraction, all DNA samples were shipped overnight to the Environmental Sample Preparation and Sequencing Facility at Argonne National Laboratory for further processing. A published PCR protocol was used to amplify the V4 region of the 16S rRNA gene, with primers designed to overlap with the Illumina sequencing primers [8,20,21]. The nucleic acid of the final product was then sequenced with an Illumina MiSeq.

### 2.5. Data Analysis and Bioinformatics

Bioinformatic analysis was completed using mothur software (https://mothur.org; accessed on 20 December 2020) [22] using a previously published protocol [23]. Sequences underwent quality control filtering, were identified using the Ribosomal Database Project classifier, clustered at the genus level (97% similarity), then binned into phylotypes [23,24]. The relative abundance of predominant taxa was calculated. Diversity, richness and evenness were measured with the inverse Simpson’s, Chao’s and Shannon’s evenness indices, respectively [8,20,21] and comparison between groups was performed using a one-way ANOVA test by time with Tukey–Kramer adjustment for multiple comparisons or the Steel–Dwass all pairs test depending on the normality of data. Community membership (an ecological measure used to compare the presence and absence of species between microbial populations) and structure (an ecological measure used to compare the proportions of shared and unshared species between microbial populations) were measured with the Jaccard and Yue and Clayton indices, respectively. Analysis of molecular variance (AMOVA) was used to assess differences between time points [8,20,21,25]. Bonferroni correction was used to adjust *p* values for multiple comparisons [20]. Clustering by timepoints was visualized with principal coordinate analyses (PCoA) and dendrograms (trees) were created and compared. Linear discriminant analysis effect size (LEfSe) was used to identify differentially abundant bacterial taxa between timepoint based on *p* < 0.05 and a linear discriminant analysis (LDA) score > 2.0. The number of different meta-communities (distinct groups of samples with similar microbial composition) that the data could be clustered into was determined using the Dirichlet multinomial mixture model (DMM) [26].

## 3. Results

### 3.1. 16S rRNA Gene Sequencing Analysis

A total of 11,875,296 reads were obtained with a mean of 141,372 reads per fecal sample (standard deviation, 52,028; median, 137,730; range, 51,902 to 475,703). A random subsample of 50,000 reads per sample was used to normalize data. The obtained coverage for all samples was 99.9%; therefore, subsampling was considered adequate.

### 3.2. Alpha Diversity

**Figure 1:**
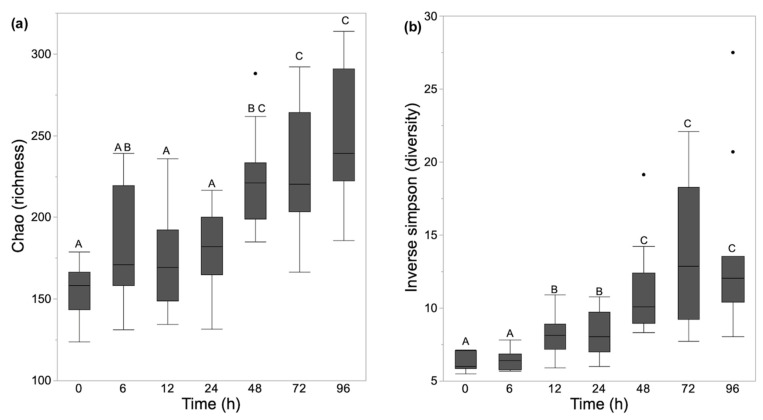
Alpha diversity measurements of the equine fecal samples stored at ambient temperature for 0 h to 96 h before freezing and sample processing represented by box and whisker plots. (**a**) Richness (Chao-1 index); (**b**) Diversity (Inverse Simpson’s diversity index). The time points that are marked with the same letter or letters (A, B, or C) had no statistically significant differences between groups (*p* > 0.05). The center line denotes the median value (50th percentile), while the upper and lower bounds of each box represent the 75th and 25th percentiles, respectively. The whiskers mark the 95th and 5th percentiles. Outliers are denoted with dots (·).

Richness increased significantly over time with samples stored at room temperature for greater than 24 h having higher richness compared with those frozen at time 0 h (*p* < 0.05; 0 h vs. 48 h, 72 h, 96 h). Evenness and diversity were similar in samples from 0 h and 6 h, but significant differences were identified between samples frozen at 0 h and those stored for 12 h or more at room temperature (*p* < 0.05; 0 h vs. 12 h, 24 h, 48 h, 72 h, 96 h) (Figure 1).

### 3.3. Relative Abundance and LEfSe Analysis

**Figure 2:**
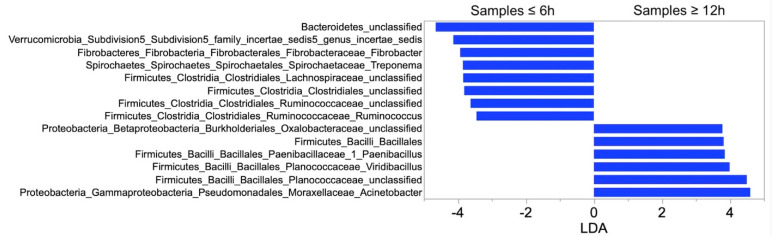
Linear discriminant analysis (LDA) scores of differentially enriched taxa in fecal samples stored at ambient temperature for 0 h to 6 h (left panel) and 12 h to 96 h (right panel) before freezing and sample processing.

Overall, a total of 27 phyla were detected with Bacteroidetes, Firmicutes and Proteobacteria comprising 73% of the total sequences at the phylum level. Fifty-seven different classes, 99 orders and 220 families were identified, but only 11 of 57, 12 of 99 and 17 of 220 each accounted for ≥0.1% of sequences overall, respectively. Overall, 605 genera were detected. Eighty-three of those were present at relative abundance of >0.05% and 12 at relative abundance of >1%. LEfSe analysis identified enriched taxa in samples frozen at each timepoint (Table 2). A significant increase in the number of taxa belonging to Proteobacteria phylum was observed in samples frozen at 72 h and 96 h compared with other time points (Table 2). LEfSe analysis showed an enrichment of Fibrobacter and Ruminococcus in samples from 0 h and 6 h compared to later samples; whereas the genera Planococcaceae (*incertae sedis*), Acinetobacter and unclassified genera of the families Bacillaceae and Enterobacteriaceae were enriched in samples stored at room temperature for 24 h or greater (Table 2, Figure 2).

### 3.4. Community Membership and Structure

**Figure 3:**
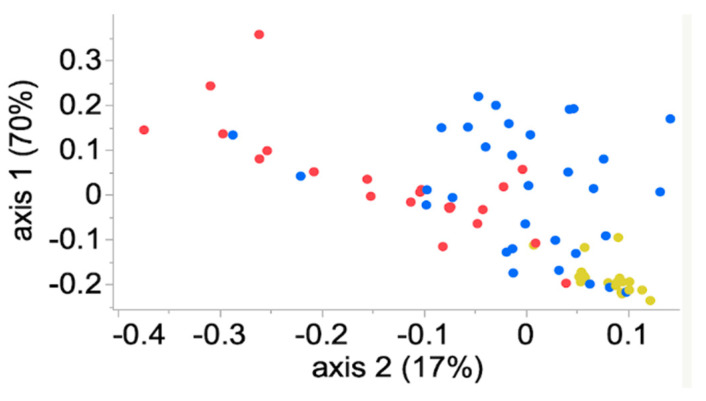
Scatter plot of the community membership (Jaccard index) of the equine fecal microbiota stored at ambient temperature for 0 h to 6 h (yellow), 12 h to 48 h (blue) and 72 h to 96 h (red) before freezing and sample processing.

**Figure 4:**
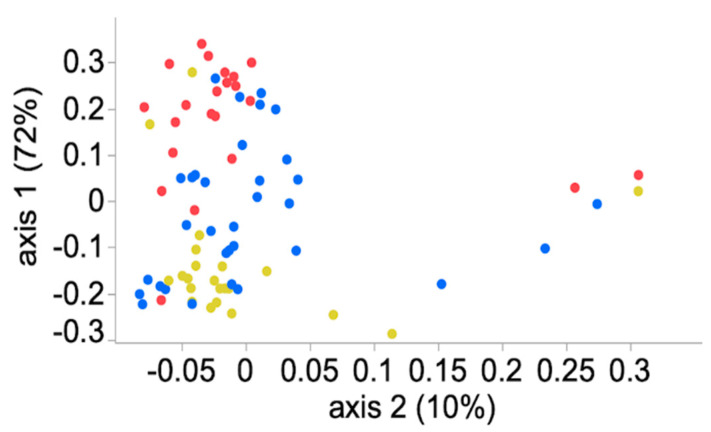
Scatter plot of the community structure (Yue and Clayton index) of the equine fecal microbiota stored at ambient temperature for 0 h to 6 h (yellow), 12 h to 48 h (blue) and 72 h to 96 h (red) before freezing and sample processing.

Samples frozen within the first 12 h after collection shared similar community membership (Jaccard index; AMOVA, *p* > 0.05; 0 h vs. 6 h, 12 h). The community structure (Yue and Clayton index) was similar for samples collected at 0 h and 6 h, but it was different between samples frozen at 0 h and 12 h or greater (AMOVA, *p* < 0.05; 0 h vs. 12 h, 24 h, 48 h, 72 h, 96 h) (Figure 3 and Figure 4).

### 3.5. Meta-Communities Analyses

Using the DMM, three meta-communities were identified; the first group comprised 100% (11/11) samples frozen at 0 h, 100% (8/8) of samples frozen at 6 h and 73% (8/11) at 12 h. The second group was comprised by 73% (8/11) of samples frozen at 24 h and 64% (7/11) of samples frozen at 48 h. The third group comprised 82% (9/11) fecal samples frozen at 96 h and 64% (7/11) of samples frozen at 72 h.

## 4. Discussion

This study aimed to investigate the effect of sample storage at room temperature for up to 96 h on the equine fecal microbiota composition. Results from the present study revealed that storage of equine fecal samples at room temperature for over 6 h had a demonstrable effect on the alpha- and beta-diversity of the microbiota. The DMM showed that all samples from 0 h and 6 h were similar indicating a significant microbial variability between those samples and samples stored at room temperature for 12 h or greater. These results are in agreement with two previous studies in horses documenting that storage of equine fecal samples at ambient temperature between 10° to 32° Celsius for more than 6 h has a significant effect on the alpha- and beta-diversity of the fecal microbiota [14,15]. These findings indicate that when ultra-low temperature storage conditions are unavailable for immediate freezing, alteration of the bacterial composition should be minimized by freezing samples within 6 h after collection.

LEfSe analysis showed that genera from the family Fibrobacteraceae (*Fibrobacter*) and Ruminococcaceae (*Ruminococcus*) were enriched in samples from 0 h and 6 h compared to later samples; whereas taxa from the families Bacillaceae, Planococcaceae, Enterobacteriaceae and Moraxellaceae were found to be more abundant in samples stored at room temperature greater than 24 h. Consistent with previous studies in horses, Fibrobacteraceae and Ruminococcaceae decreased significantly in fecal samples from horses stored at room temperature for 12 h compared with fresh samples [14]. In humans, Ruminococcus also decreased significantly in fecal samples stored at room temperature for 12 h compared to fresh samples [3,27]. Taxa belonging to the families Bacillaceae, Planococcaceae, Enterococcaceae, Enterobacteriaceae and Moraxellaceae were enriched in all samples from 12 h compared with samples stored at ambient temperature for less than 6 h. In fact, taxa from the family Planococcaceae and Moraxellaceae were not identified in fresh samples. In humans, taxa belonging to the same families were found to be more abundant in samples stored at room temperature [28]. Fibrobacteraceae and Ruminococcaceae are anaerobic bacteria, whereas Bacillaceae, Planococcaceae, Enterococcaceae, Enterobacteriaceae and Moraxellaceae are either aerobes, facultative anaerobes, or aerotolerant anaerobes [14]. Therefore, it is possible that a shift in fecal microbiome from anaerobic to aerobic bacteria occurred in samples stored at room temperature for more than 6 h. While the fecal samples in the current study were stored in sealed containers, transient exposure to oxygen was unavoidable at the time of sample transfer following initial collection from each patient and again at the time of division of samples into aliquots. A similar shift from anaerobic to aerobic bacteria was previously reported in fecal samples from cattle exposed at open field conditions for 72 h [11]. The authors of the aforementioned study hypothesized that differences in the content of oxygen between the gut and room environment can be responsible, at least in part, for the shift in the bacterial structure over time. However, neither in our study nor in the cattle study, the oxygen content in the fecal samples was measured; thus, this hypothesis remains speculative and requires further investigation.

Of interest, our results are in contrast to studies investigating the effect of room temperature fecal sample storage on the human and feline fecal microbiota. No significant differences were noted in alpha- and beta-diversity indices or relative abundance of different taxa over time between feline fecal samples stored at room temperature for up to 96 h prior to freezing and sample processing [8]. In humans, some studies showed that fecal sample storage at ambient temperature for up to 24 h did not lead to significant changes in fecal microbial composition when compared to direct freezing [13]. In another human study, room temperature storage for up to 14 days had minimal effect on community structure and the relative abundance of different taxa in fecal samples [29]. While the underlying cause of these inter-species inconsistencies in the fecal microbial community stability over time is unknown, it can be in part attributed to dietary differences altering the fecal bacterial and metabolic composition.

There are several limitations to the current study. This study only assesses the equine fecal microbiota in fecal samples stored at the seven specific time points for up to 96 h at ambient temperature before freezing. No conclusions can be made for samples remaining at ambient temperature for a longer duration nor for samples stored in other conditions, such as refrigeration at 4 °C or suspension in preservative solution (e.g., Tris-EDTA or RNAlater). Canine and feline samples stored for up to 14 days in a refrigerator at 4 °C were stable with minimal impact on the fecal microbiota. Additional studies are needed to determine if refrigeration confers a similar microbial stability to equine fecal samples [30].

## 5. Conclusions

In conclusion, storage of equine fecal samples at ambient temperature for up to 6 h before freezing following sample collection had minimal effect on the microbial composition and these samples had similar alpha- and beta-diversity and relative abundance of predominant taxa when compared to fecal samples immediately frozen at −80 °C. Longer-term storage at ambient temperature resulted in alterations in alpha-diversity, community membership and structure and the relative abundance of predominant taxa. When ultra-low temperature storage conditions are unavailable for immediate freezing, equine fecal samples should be frozen within 6 h after collection to minimize storage induced alterations in bacterial composition. In order to better establish appropriate storage protocols (>6 h after sample collection) for equine fecal microbiota field studies, further investigation into commercial DNA preservation solutions or short-term refrigeration is warranted.

## Figures and Tables

**Table 1:** Study population signalment and weight.

Horse	Breed	Age (year)	Sex	Weight (kg)
1	Thoroughbred	12	F	581
2	Thoroughbred	12	M	543
3	Thoroughbred	5	M	536
4	Thoroughbred	5	M	529
5	Thoroughbred	5	M	572
6	Thoroughbred	11	F	514
7	Thoroughbred	4	F	481
8	Thoroughbred	9	M	618
9	Thoroughbred	8	F	508
10	Thoroughbred	5	F	505
11	Thoroughbred	12	F	492

F = female (mare), M = castrated male (gelding).

**Table 2:** Differentially enriched taxa with linear discriminant analysis (LDA score ≥ 2 identified in the horse’s fecal samples storage at ambient temperature up to 96 h.

Time (Hours)	Phylum	Genus	LDA
0	Bacteroidetes	*Bacteroidetes* (unclassified)	4.86
		*Bacteroidales* (unclassified)	3.98
	Fibrobacteres	*Fibrobacter*	4.11
	Firmicutes	*Lachnospiraceae* (unclassified)	4.11
		*Firmicutes* (unclassified)	3.67
		*Clostridia* (unclassified)	3.43
		*Pseudobutyrivibrio*	3.11
		*Cellulosilyticum*	2.24
	Proteobacteria	*Vampirovibrio*	2.73
	Spirochaetes	*Treponema*	4.09
	Verrucomicrobia	*5 genus (incertae sedis)*	4.44
6	Firmicutes	*Clostridiales* (unclassified)	3.99
		*Ruminococcus*	3.65
		*Saccharofermentans*	2.89
		*Weissella*	2.65
	Lentisphaerae	*Victivallis*	2.80
	Verrucomicrobia	*Verrucomicrobia* (unclassified)	2.81
12	Firmicutes	*Acidaminococcaceae* (unclassified)	3.30
	Tenericutes	*Anaeroplasma*	3.00
24	Firmicutes	*Caryophanon*	3.20
	Proteobacteria	*Sphingomonas*	4.70
48	Actinobacteria	*Arthrobacter*	2.97
		*Micrococcaceae* (unclassified)	2.78
		*Cellulosimicrobium*	2.59
	Firmicutes	*Bacillales unclassified*	4.23
		*Viridibacillus*	4.19
		*Rummeliibacillus*	3.86
		*Kurthia*	3.70
		*Lysinibacillus*	3.41
	Proteobacteria	*Acinetobacter*	4.78
72	Actinobacteria	*Nocardioides*	3.04
		*Micromonospora*	2.17
	Firmicutes	*Paenibacillus*	4.15
		*Tumebacillus*	3.88
		*Cohnella*	3.72
		*Brevibacillus*	2.50
	Proteobacteria	*Oxalobacteraceae* (unclassified)	4.12
		*Sphingomonas*	4.03
		*Rhizobiales* (unclassified)	4.02
		*Sandarakinorhabdus*	3.87
		*Enterobacteriaceae* (unclassified)	3.52
		*Alcaligenaceae* (unclassified)	2.87
		*Sphingopyxis*	2.03
96	Actinobacteria	*Leifsonia*	3.19
		*Actinomycetales* (unclassified)	2.73
		*Coriobacteriaceae* (unclassified)	2.49
		*Sanguibacter*	2.28
	Bacteroidetes	*Sphingobacterium*	3.14
		*Segetibacter*	2.89
	Firmicutes	*Clostridium_sensu_stricto*	3.23
		*Sedimentibacter*	3.13
		*Bacillaceae 1* (unclassified)	2.92
		*Planococcaceae (incertae sedis)*	2.58
		*Mogibacterium*	2.56
		*Peptococcaceae 1* (unclassified)	2.34
		*Clostridiaceae 1* (unclassified)	2.22
		*Parasporobacterium*	2.18
		*Sporobacter*	2.14
		*Bacillus*	2.12
		*Desulfitobacterium*	2.08
	Proteobacteria	*Brevundimonas*	3.92
		*Xanthomonadaceae* (unclassified)	3.81
		*Massilia*	3.41
		*Burkholderiales* (unclassified)	3.33
		*Pseudomonas*	3.21
		*Sphingomonadaceae* (unclassified)	3.14
		*Azospirillum*	3.14
		*Magnetospirillum*	3.08
		*Ensifer*	3.06
		*Pseudomonadaceae* (unclassified)	3.02
		*Myxococcales* (unclassified)	2.71
		*Devosia*	2.25
		*Hyphomicrobium*	2.10
	TM7	*TM7 (incertae sedis)*	2.33

## Data Availability

Publicly available datasets were analyzed in this study. This data can be found at: NCBI database, bioproject number 9262978.
